# Capturing local compositional fluctuations in NMR modelling of solid solutions

**DOI:** 10.1039/d5sc04550a

**Published:** 2025-09-16

**Authors:** Ricardo Grau-Crespo, Said Hamad, Salvador R. G. Balestra, Ramsey Issa, Taylor D. Sparks, Arantxa Fernandes, Ben L. Griffiths, Robert F. Moran, David McKay, Sharon E. Ashbrook

**Affiliations:** a School of Engineering and Materials Science, Queen Mary University of London London E1 4NS UK r.grau-crespo@qmul.ac.uk; b Department, of Chemistry, University of Reading Whiteknights, Reading RG1 6DX UK; c Departamento de Sistemas Físicos, Químicos y Naturales, Universidad Pablo de Olavide Ctra. Utrera Km. 1 Sevilla 41013 Spain; d Departamento de Física Atómica, Molecular y Nuclear, Área de Física Teórica, Universidad de Sevilla Av. Reina Mercedes s/n 41012 Seville Spain; e Department of Materials Science and Engineering, University of Utah Salt Lake City UT 84112 USA; f School of Chemistry, EaStCHEM and Centre of Magnetic Resonance North Haugh St Andrews KY16 9ST UK sema@st-andrews.ac.uk; g EPCC, Bayes Centre, University of Edinburgh 47 Potterrow Edinburgh EH8 9BT UK

## Abstract

Understanding the atomic-scale local properties of solid solutions is crucial for deciphering their structure–property relationships. In this work, we present a computational approach that combines solid-state nuclear magnetic resonance (NMR) spectroscopy with density functional theory (DFT) calculations to investigate local chemical environments in solid solutions. Previous canonical ensemble models, which only sample configurations at a fixed composition of the simulation cell, fail to capture local compositional fluctuations that can significantly influence the NMR spectra. To address this limitation, we employ a grand-canonical ensemble approach enabling a more comprehensive representation of the contributions of all possible local chemical environments to the NMR spectrum, using a La_2_(Zr_1−*x*_Sn_*x*_)_2_O_7_ pyrochlore solid solution as a case study. To mitigate the high computational cost of such simulations, we also explore ensemble truncation strategies and the use of machine learning (ML) to aid predictions of NMR chemical shifts, achieving a significant reduction in computational cost while maintaining most of the predictive power. Our results show that combining the grand-canonical approach with machine learning and ensemble truncation offers an efficient framework for modelling and interpreting NMR spectra in disordered crystalline materials.

## Abstract 2

Density functional theory calculations in a grand-canonical configurational ensemble allow accurate modelling of NMR spectra of solid solutions.

## Graphical abstract

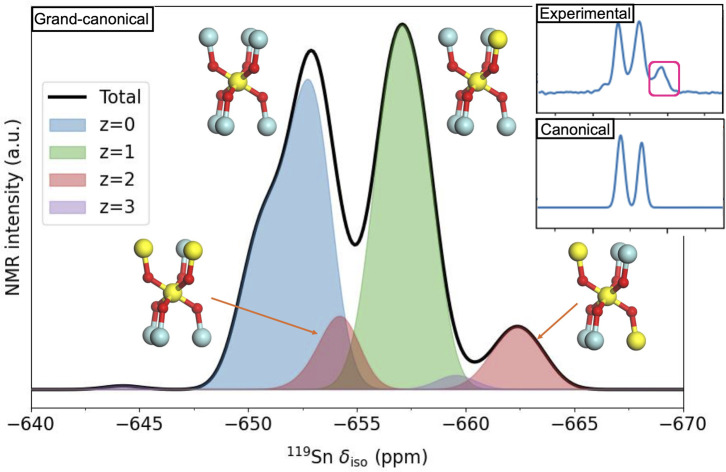

## 1 Introduction

The controlled compositional variation in solid solutions offers extensive possibilities for tuning the physical and chemical properties of functional materials, making such materials pivotal in many technological applications, from electronics to energy storage and conversion.^1^ A fundamental challenge, however, lies in accurately characterising and understanding the local structural environments arising from compositional disorder. While X-ray diffraction and other long-range averaged techniques provide invaluable information about the global crystal structure, they fail to resolve the subtle local variations and compositional fluctuations that significantly influence properties such as ionic conductivity, catalytic activity, and electronic structure. In contrast, solid-state nuclear magnetic resonance (NMR) spectroscopy has emerged as an indispensable technique for probing local atomic environments, thanks to its intrinsic sensitivity to short-range structural variations without requiring long-range periodicity.^2,3^

Although the theory to obtain NMR chemical shifts from quantum-mechanical simulations in a periodic solid is well established,^4–6^ the interpretation of NMR spectra from disordered solid solutions remains highly challenging, primarily because spectra represent statistical averages over numerous distinct chemical environments. Ensemble-based approaches combining density functional theory (DFT) calculations with statistical mechanics have demonstrated considerable promise in simulating NMR spectra of site-disordered solids and interpreting the complex spectral lineshapes obtained.^7–9^ Such approaches usually involve systematically enumerating chemical configurations within a symmetry-adapted configurational ensemble, allowing for statistical averaging of computed chemical shifts to reconstruct the experimental NMR spectrum (usually assuming this is acquired under magic-angle spinning (MAS) conditions). Despite their success, the methods used in previous studies face two significant limitations: firstly, finite supercell sizes restrict the range of possible local chemical environments; secondly, these approaches are computationally demanding, rapidly becoming impractical as the configurational space expands.

Here, we propose a computational strategy based on grand-canonical ensembles (as in the so-called quasi-chemical approximation)^10^ to overcome these challenges. In contrast to canonical-ensemble methods used before for solid-state NMR modelling, where the simulation supercell has the same composition as the solid, the grand-canonical ensemble enables the sampling of configurations with varying compositions. This flexibility provides comprehensive access to the entire range of local chemical environments, including those involving large compositional fluctuations away from the average composition of the solid, without the need for a very large simulation supercell, which is problematic due to the high computational cost of DFT evaluations of NMR chemical shifts. We illustrate the utility of this method by applying it to interpret the complex lineshapes seen in the ^119^Sn MAS NMR spectra of a La_2_(Zr_1−*x*_Sn_*x*_)_2_O_7_ pyrochlore solid solution. Pyrochlore solid solutions provide a useful test case, combining intricate local structural variations with detailed (and typically multinuclear) experimental NMR data to benchmark computational approaches.^5,7,11,12^ Moreover, we address the critical computational bottleneck associated with extensive DFT-based configurational sampling by controlled ensemble truncations and by integrating machine-learning (ML) techniques.

## 2 Methods

### 2.1. Computational methods

#### 2.1.1 Configurational ensembles

**Fig. 1:**
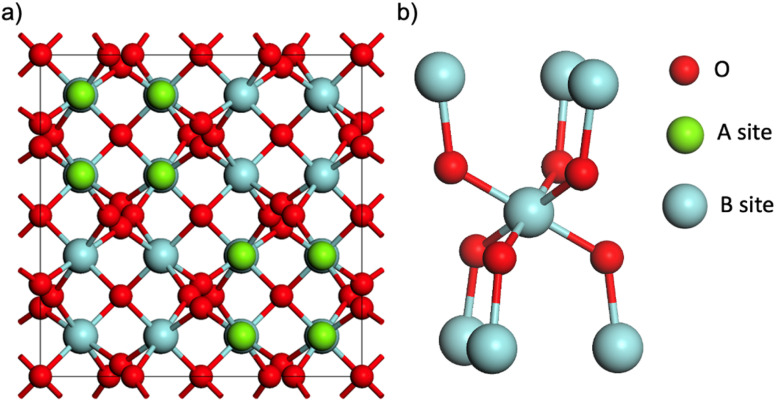
(a) Schematic showing cubic unit cell of the A_2_B_2_O_7_ pyrochlore structure. (b) Expansion showing the coordination environment of the B-site cation in the pyrochlore structure, showing the 6 next-nearest neighbouring B sites.

The A_2_B_2_O_7_ pyrochlore structure is derived from a fluorite (AO_2_) supercell, where 1/8 of the anions are removed in an ordered manner.^13–15^ This arrangement creates an eight-coordinate A site, occupied in this study by La^3+^, and a six-coordinate B site, in which Sn^4+^ and/or Zr^4+^ are distributed, as illustrated in Fig. 1.

We systematically generate all the symmetrically inequivalent configurations for each number *n* of B-site substitutions in a pyrochlore unit cell La_16_Zr_16−*n*_Sn_*n*_O_56_, using the Site Occupancy Disorder (SOD) program.^16,17^ The total number of configurations with *n* Sn substitutions:1
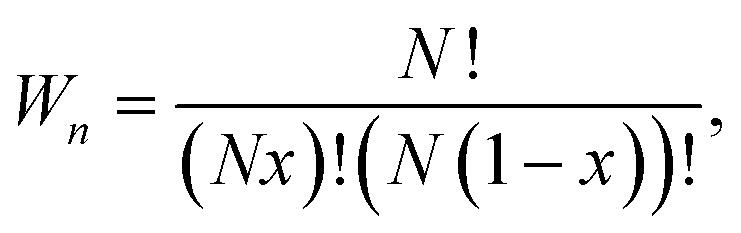


and the number of those that are inequivalent (*M*_*n*_) are listed in Table 1, for each *n*. Here *N* is the number of sites over which substitutions are considered (*i.e.*, *N* = 16 for the B sites in the pyrochlore cell) and *x* = *n*/*N* is the molar fraction of substitutions.

**Table 1:** Total number of atomic configurations (*W*_*n*_) and the number of symmetry inequivalent configurations (*M*_*n*_) in a cell with La_16_Sn_*n*_Zr_16−*n*_O_56_ composition

Chemical formula	*x*	*n*	*W*_*n*_	*M*_*n*_
La_2_Zr_2_O_7_	0	0	1	1
La_2_(Sn_0.0625_Zr_0.9375_)_2_O_7_	0.0625	1	16	1
La_2_(Sn_0.125_Zr_0.875_)_2_O_7_	0.125	2	120	3
La_2_(Sn_0.1875_Zr_0.8125_)_2_O_7_	0.1875	3	560	8
La_2_(Sn_0.25_Zr_0.75_)_2_O_7_	0.25	4	1820	22
La_2_(Sn_0.3125_Zr_0.875_)_2_O_7_	0.3125	5	4368	35
La_2_(Sn_0.375_Zr_0.625_)_2_O_7_	0.375	6	8008	65
La_2_(Sn_0.4375_Zr_0.5625_)_2_O_7_	0.4375	7	11 440	82
La_2_(Sn_0.5_Zr_0.5_)_2_O_7_	0.5	8	12 870	97
La_2_(Sn_0.5625_Zr_0.4375_)_2_O_7_	0.5625	9	11 440	82
La_2_(Sn_0.625_Zr_0.375_)_2_O_7_	0.625	10	8008	65
La_2_(Sn_0.875_Zr_0.3125_)_2_O_7_	0.6875	11	4368	35
La_2_(Sn_0.75_Zr_0.25_)_2_O_7_	0.75	12	1820	22
La_2_(Sn_0.8125_Zr_0.1875_)_2_O_7_	0.8125	13	560	8
La_2_(Sn_0.875_Zr_0.125_)_2_O_7_	0.875	14	120	3
La_2_(Sn_0.9375_Zr_0.0625_)_2_O_7_	0.9375	15	16	1
La_2_Sn_2_O_7_	1	16	1	1
**Total**			**65 536**	**531**

The thermodynamics of mixing and the NMR spectra in this system were simulated using a grand-canonical ensemble approach, which includes configurations of different compositions with probabilities that depend on the relative chemical potential of the two species being mixed. Whereas the canonical ensemble only considers configurations with *n* = *xN* substitutions, the grand-canonical ensemble considers configurations with all possible compositions *n* = 0, …, *N*. The probability of the *m*th configuration with *n* substitutions in the grand-canonical ensemble is:2
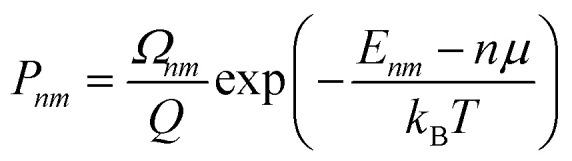
where *μ* is the chemical potential of the substitution (more precisely, the difference between chemical potentials of the two species), *Ω*_*nm*_ is the degeneracy of the configuration, and *Q* is the grand-canonical partition function. If we want to model a particular composition *x* using the grand-canonical ensemble, we can easily find (by solving a polynomial equation, see *e.g.* ref. 18) the value of *μ* that corresponds to that composition, since:3
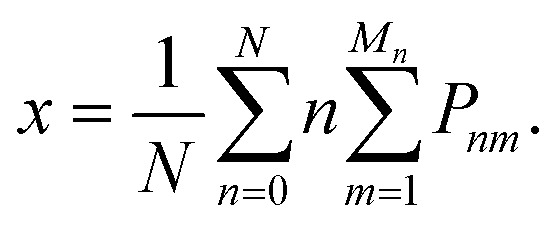


In the limit of full disorder (formally infinite temperature), it is not necessary to calculate a chemical potential to obtain the probabilities, as in that case it can be demonstrated that:

4 *P*_*nm*_ = *Ω*_*nm*_*x*^*n*^(1 − *x*)^*N*−*n*^.

**Fig. 2:**
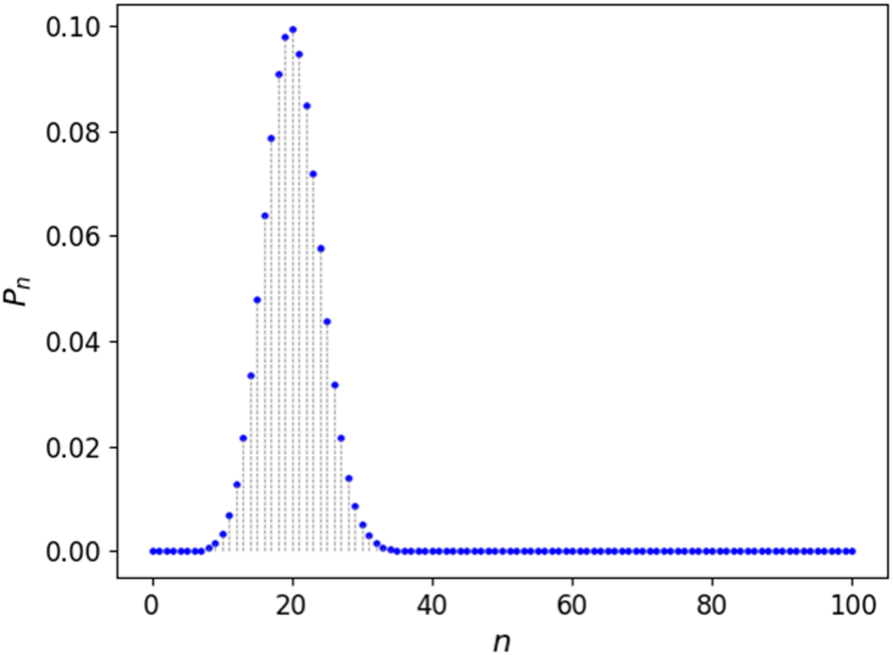
Cumulative probability *P*_*n*_ of all configurations with *n* substitutions in a hypothetical supercell with *N* = 100 sites, as a function of *n*, when the overall fraction of substitutions is *x* = 0.2.

The dependence on *x* in the equation above is such that the largest contributions, in terms of occurrence probabilities, come from configurations with *n* close to *xN*. The cumulative probabilities of all configurations with *n* substitutions are:5
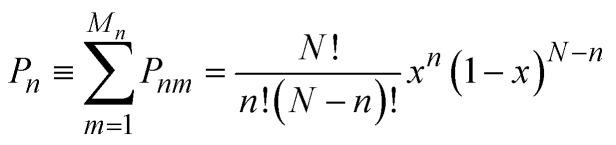


which has a maximum when *n* = *xN*. For example, Fig. 2 shows the result obtained if we take a hypothetical large cell with *N* = 100 and model the composition *x* = 0.2. Since in this large cell the canonical ensemble with *n* = *xN* (*n* = 20 in this case) is likely to contain most meaningful chemical environments, the canonical ensemble works well here as a first approximation. However, in the case of much smaller cells (such as those often required when predicting NMR parameters with periodic DFT), the canonical ensemble may be a poor representation of the disordered solid, as it can miss possible local environments and/or fail to reproduce the correct statistical weights associated with each configuration.

The grand-canonical algorithm used here has been implemented within the SOD code.^16^ It is useful to note that, in the context of solid solutions (and in other mixtures), the ensemble described above is often called the semi-grand-canonical ensemble,^19,20^ to emphasise that it is only the relative proportion between the two species that is allowed to change, while the total number remains constrained by the number of available crystal sites. We prefer to refer to this ensemble simply as grand-canonical, as this more general term includes the case when some of the species disordered over the crystal sites are vacancies or interstitials, *e.g.* H distribution in equilibrium with an external H_2_ gas, which can be treated within the same formalism.^21,22^

#### 2.1.2 DFT calculations

We conducted geometry optimisations for the 531 independent configurations at DFT level using the CASTEP code.^6,23^ We employed the PBE exchange–correlation functional^24^ with ultrasoft pseudopotentials^25^ to account for core-valence interactions, incorporating scalar relativistic effects *via* ZORA.^26^ A planewave energy cutoff of 60 Ry (∼816 eV) was used, and the first Brillouin zone was sampled *via* a Monkhorst–Pack grid^27^ with a reciprocal space grid spacing of 0.04 2π Å^−1^. During geometry optimisation, all atomic coordinates and unit cell parameters were allowed to vary, with an energy tolerance of 10^−5^ eV per atom for geometry optimisation, and an electronic structure energy tolerance of 10^−9^ eV per atom; such tight tolerances were required to obtain fully-relaxed geometries.^7^

The computation of NMR parameters used the DFT settings described above and employed the gauge-including projector augmented wave (GIPAW) approach^6,23^ to reconstruct the all-electron wave functions in the presence of an induced magnetic field. On average, each optimisation + NMR calculation of a configuration required ∼90 minutes using 896 cores on ARCHER2,^28^ or around 40 million CPU hours for all configurations. The calculations produced the absolute shielding tensor (*σ*^calc^) in the crystal frame. Diagonalising the symmetric part yielded the principal components *σ*^calc^_11_, *σ*^calc^_22_ and *σ*^calc^_33_, from which we can determine the isotropic shielding constant, *σ*^calc^_iso_ = (*σ*^calc^_11_ + *σ*^calc^_22_ + *σ*^calc^_33_)/3. To allow comparison with experimental data, we derived the corresponding isotropic chemical shift, *δ*^calc^_iso_, using *σ*^calc^_iso_.

For each configuration we generated the predicted ^119^Sn MAS NMR spectra by including an individual Gaussian function for each *δ*^calc^_iso_ value obtained in the calculation of the corresponding configuration. A fixed line broadening of 0.85 ppm was applied to each Gaussian function, which was chosen based on the linewidth seen for the single signal in the ^119^Sn MAS NMR spectrum of the end member La_2_Sn_2_O_7_. The spectra were then weighted by the respective probability of that configuration before being combined to generate a final spectrum for each composition.

#### 2.1.3 Machine learning

To explore ways to accelerate the NMR simulations, we used ML models, trained on DFT data, for predicting the ^119^Sn isotropic chemical shifts. As features to describe the chemical environment around the Sn atoms, we used sixth-order local-averaged Steinhardt parameters,^29,30^ obtained here from the unrelaxed configuration geometries (because we want the ML model to predict the chemical shifts without the need for a DFT geometry relaxation). The descriptors, a combination of 4 local and 6 global features, were calculated using the PLUMED library (we used LOCAL_Q6 and COORDINATIONNUMBER),^31^ and the code to extract those from the configurations is available. Correlated features were eliminated (global features corresponding to the average Sn–Sn and Sn–Zr NNN coordination are linearly correlated). We then tested several classical ML models, from linear regression to gradient-boosted methods. Because the goal is to reduce the number of configurations to be calculated at DFT level, we created training and test sets based on configurations, rather than on data points, *i.e.*, in the test set we do not use any chemical shifts of atoms belonging to configurations used in the training set.

### 2.2 Experimental methods

#### 2.2.1 Synthesis and basic characterisation

La_2_(Sn_*x*_Zr_1−*x*_)_2_O_7_ samples were prepared for compositions ranging from *x* = 0 to 1 in increments of 0.125 using a conventional solid-state reaction method, described in ref. 32. Commercially available La_2_O_3_ (Sigma Aldrich, 99.9%), SnO_2_ (Sigma Aldrich, 99.9%), and ZrO_2_ (Sigma Aldrich, 99%) were preheated for 10 h at 800 °C to remove moisture and surface hydroxides before being weighed in stoichiometric ratios. The precursors were ball-milled using ZrO_2_ media with acetone/isopropanol as the milling solvent for 1 h at 600 rpm to achieve uniform mixing and reduce particle size. The resulting powder was pressed into pellets, placed in alumina boats, and sintered in a tube furnace at ∼1673 K for 24 h. The sintered pellets were reground and re-pressed before undergoing a second firing under the same conditions for an additional 24 h. Phase purity and crystallinity of the samples were assessed using a PANalytical Empyrean X-ray diffractometer with CuK_α1_ radiation (*λ* = 1.5406 Å) and a X'celerator RTMS strip detector. Diffraction data were collected over a 2*θ* range of 10 to 80° or 100° with a step size of 0.02° and a counting time of 0.4 s per step. Powder XRD analysis confirmed the formation of a single-phase pyrochlore structure across all compositions.

#### 2.2.2 Solid-state NMR spectroscopy

The ^119^Sn MAS NMR spectra were acquired using a Bruker Avance III 400 MHz NMR spectrometer, equipped with a 9.4 T magnet, operating at a Larmor frequency of 149.2 MHz for ^119^Sn. Powdered samples were packed into 4 mm ZrO_2_ rotors and spun at 14 kHz using a 4 mm HX probe. Spectra were acquired using a spin-echo pulse sequence, with a radiofrequency field strength of ∼111 kHz (π/2 ≈ 2.25 μs), averaging between 128 and 8720 transients with a recycle interval of 30 s. Chemical shifts are reported in ppm relative to (CH_3_)_4_Sn as a primary reference, measured using a secondary reference of SnO_2_ (*δ*_iso_ = −604.3 ppm).^33^

## 3 Results and discussion

### 3.1 Mixing thermodynamics

**Fig. 3:**
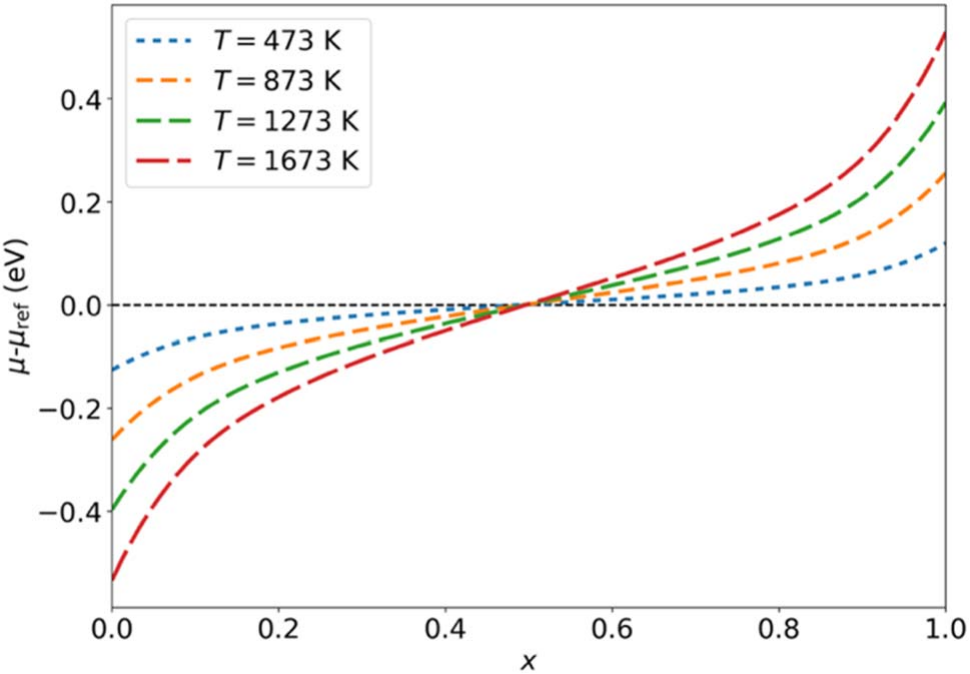
Chemical potential of the substitutions in La_2_(Sn_*x*_Zr_1−*x*_)_2_O_7_, relative to the reference chemical potential: *μ*_ref_ = (*E*[La_2_Sn_2_O_7_] − *E*[La_2_Zr_2_O_7_])/2, where the *E* values are the DFT energies per formula unit.

We initially used the DFT energies calculated from the full ensemble of configurations to examine the mixing thermodynamics of the La_2_(Sn_*x*_Zr_1−*x*_)_2_O_7_ solid solution. Fig. 3 shows how the calculated chemical potential depends on the composition, allowing the thermodynamic calculations at any value of *x* value (not only at the canonical values *x* = *n*/*N* for which we have DFT data).

The fact that the grand-canonical ensemble is a more complete representation of the disordered solid than the canonical ensemble is best illustrated by comparing the corresponding configurational entropies. For the canonical ensemble, the maximum mixing (configurational) entropy per site, obtained in the limit of full disorder (*T* → ∞), is:6
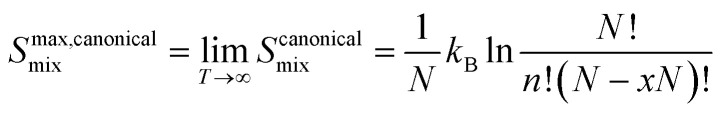


which matches the exact value:7
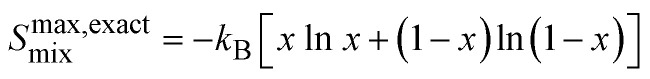


only in the limit of an infinite supercell (*N* → ∞). In contrast, it can be demonstrated that the mixing entropy obtained from the grand-canonical ensemble:8
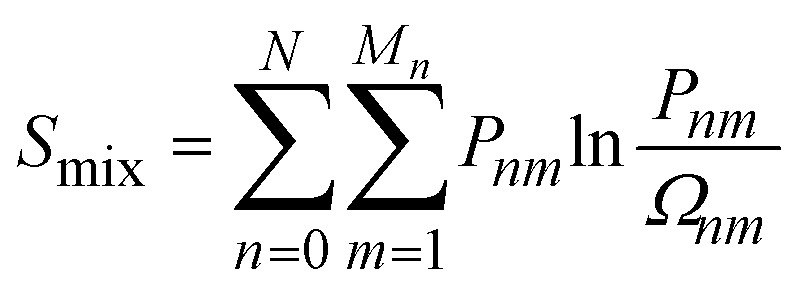


leads to the exact value for any supercell size, *i.e.*,9
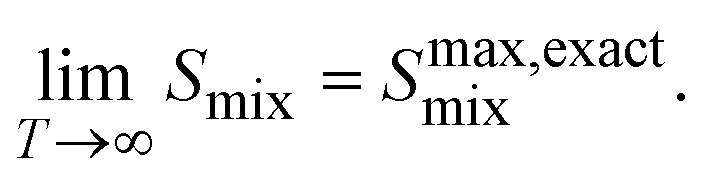


This result is a consequence of the fact that the grand-canonical ensemble admits all possible configurations of substitutions, whereas the canonical ensemble only admits the configurations that are compatible with the canonical composition within a cell, an observation that is important for our discussion of the ^119^Sn MAS NMR spectra below.

**Fig. 4:**
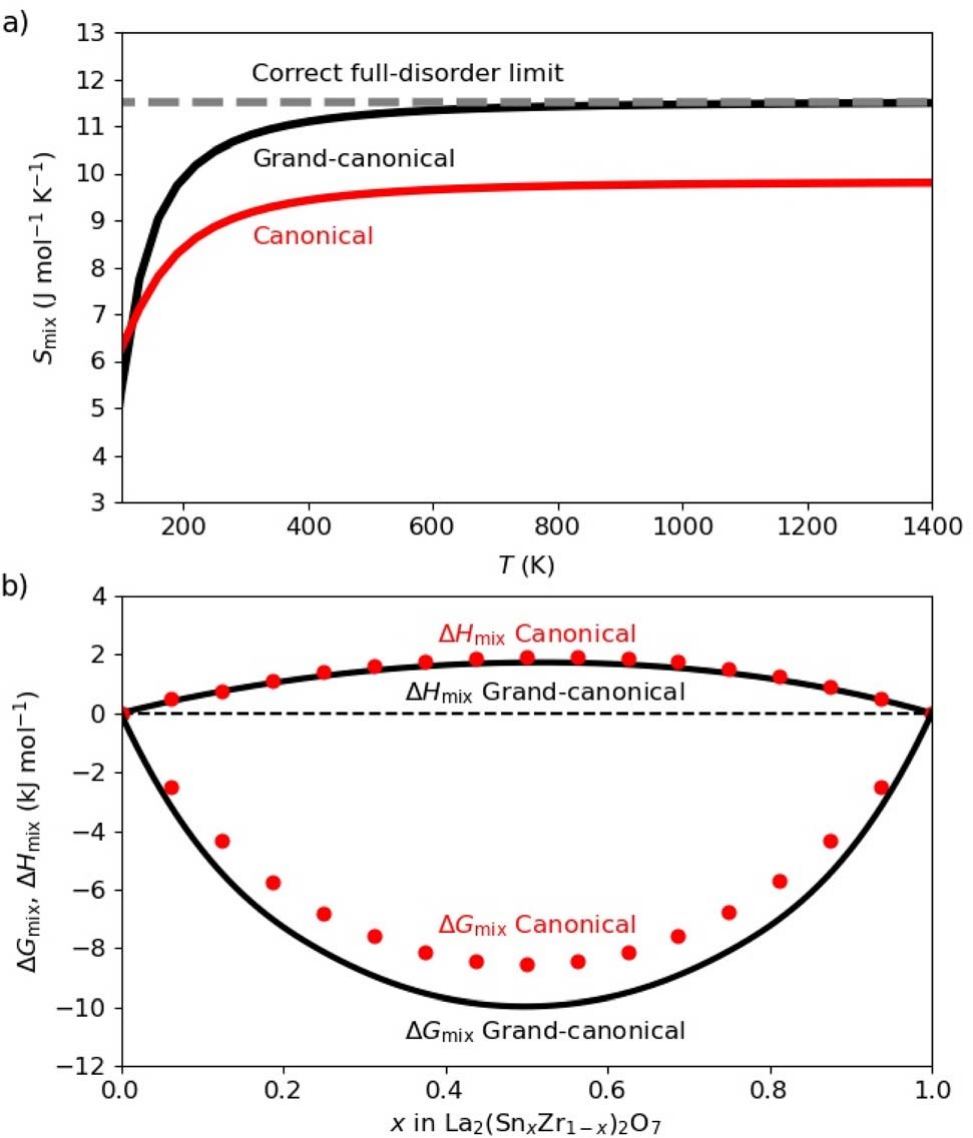
(a) Variation of the calculated configurational entropy *S*_mix_(per formula unit) with temperature in the La_2_(Sn_*x*_Zr_1−*x*_)_2_O_7_ solid solution. (b) Mixing enthalpies (Δ*H*_mix_) and free energies (Δ*G*_mix_), per formula unit, calculated at 873 K. Canonical and grand-canonical ensembles are represented by red and black colours, respectively, in both plots. Canonical values for Δ*H*_mix_and Δ*G*_mix_are represented as dots because they are directly evaluated at discrete compositions *x* = *n*/*N*.

We now illustrate the performance of the grand-canonical ensemble model in the case of the La_2_(Sn_*x*_Zr_1−*x*_)_2_O_7_ solid solution and compare this with the canonical ensemble model. Fig. 4a shows the configurational entropy (calculated from the DFT energies) for *x* = 0.5, as a function of temperature, for each ensemble. The maximum entropy (in the limit of infinite temperature) per formula unit that can be obtained from the canonical model in a cell with 8 formula units corresponds to 
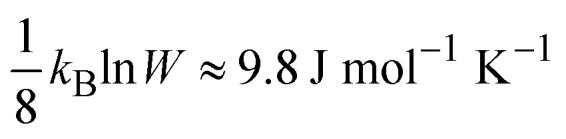
, where *W* = 16!/(8!8!) = 12 870 is the total number of configurations with that composition in the cell. However, the exact maximum entropy should be 2*k*_B_ ln 2 ≈ 11.5 J mol^−1^ K^−1^, because there are two B sites per La_2_(Sn_0.5_Zr_0.5_)_2_O_7_ formula unit, each with 1/2 occupancy by Sn and 1/2 occupancy by Zr in the formula unit. Therefore, the canonical model with this cell size can only give ∼85% of the possible configurational entropy for the *x* = 0.5 composition. This can be interpreted as the canonical model missing some local chemical environments/configurations, which are responsible for the unaccounted 15% of the configurational entropy. That incompleteness has important consequences for the discussion below about the agreement between calculated and experimental NMR spectra: the missing configurations in the canonical ensemble correspond mainly to less probable environments, but some of these have distinctive chemical shifts that can affect the spectra. The grand-canonical model, on the other hand, gives a better description of the entropy, including the correct high-temperature limit of the configurational entropy, and therefore leads to a more accurate thermodynamic analysis (and to a better account of possible chemical environments, as will be discussed below). At any temperature high enough for cation diffusion to take place, say above at least 600 K, the grand-canonical configurational entropy is practically identical to the configurational entropy of a perfectly disordered system.

Fig. 4b shows the calculated mixing enthalpies and free energies at *T* = 873 K, as a function of composition. Enthalpies were obtained from the average of DFT energies as 
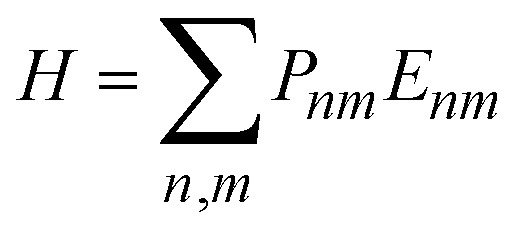
, *i.e.* neglecting pressure and vibrational effects which typically contribute little to the mixing value, Δ*H*_mix_ = *H*(*x*) − (1 − *x*)*H*(0) − *xH*(1), in oxide solid solutions.^34,35^ Whereas the canonical ensemble leads to acceptable results for the mixing enthalpy, the calculation of the mixing free energy (Δ*G*_mix_ = Δ*H*_mix_ − *TS*_mix_) is of course more problematic, due to the underestimation of the mixing entropy in the canonical ensemble.

The mixing thermodynamics analysis shows that, while the system is not an ideal solid solution, because it has a (small) positive mixing enthalpy, it does exhibit an almost perfect level of disorder for equilibration temperatures above ∼600 K. Since the synthesis procedure involves temperatures much higher than that, and the cation distribution does not equilibrate upon cooling to room temperature (due to high barriers for cation diffusion), these results suggest that the solid solution will remain very highly disordered when cooled to room temperature.

### 3.2 NMR spectroscopy: experiments

**Fig. 5:**
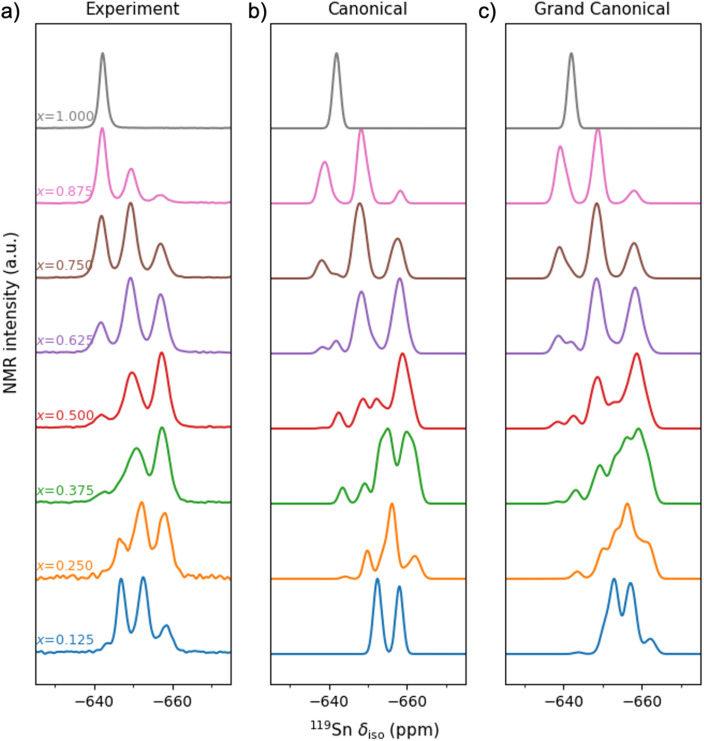
(a) Experimental and (b and c) simulated ^119^Sn (9.4 T, 14 kHz) NMR spectra of La_2_(Sn_*x*_Zr_1−*x*_)_2_O_7_ for different compositions. The (b) and (c) panels correspond to the simulated MAS NMR spectra obtained using the canonical and grand-canonical ensembles, respectively. The configurational temperature used in the averaging was 873 K.

Fig. 5a shows the experimental ^119^Sn MAS NMR spectra of La_2_(Sn_*x*_Zr_1−*x*_)_2_O_7_. As discussed earlier, each Sn in this structure is surrounded by 6 nearest-neighbour (NN) O atoms, and 6 next-nearest-neighbour (NNN) B sites, each of which can be occupied by Sn or Zr. For La_2_Sn_2_O_7_ (*i.e.*, the solid solution end-member with *x* = 1), a single signal is seen at ∼ −641 ppm, as expected from the crystal structure, which corresponds to Sn on the six-coordinate B site, surrounded by 6 Sn NNN. Interestingly, this signal is not as sharp as that seen for Y_2_Sn_2_O_7_ in previous work,^11^ where the small ^119^Sn-^117^Sn *J* coupling was resolved. This difference likely results from the interaction of ^119^Sn with ^139^La, which has both a higher *g* than ^89^Y and is quadrupolar (*I* = 7/2), leading to the presence of small but significant second-order quadrupolar-dipolar cross terms^36^ which can also be seen in ^17^O MAS NMR spectra of La_2_Sn_2_O_7_.^32^

Upon substitution of Zr, a change in isotropic chemical shift is expected, owing to the difference in electronegativity between Sn^4+^ and Zr^4+^ cations. The number of Sn NNN can vary from 0 to 6; therefore, in principle, 7 signals might be expected in the spectrum. However, when 2, 3 or 4 Sn/Zr are substituted, there are 3 different ways of arranging these in each case, leading to a total of 13 different NNN environments, although the shift differences between varying arrangements of the same number of Sn/Zr may be smaller and less well resolved in the spectrum. Fig. 5a shows that as Zr is substituted into Y_2_Sn_2_O_7_ (*e.g.*, *x* = 0.875), additional signals are seen at decreasing shift (−649 and −657 ppm), which could be assumed to result from Sn with 1 and 2 Zr NNN, respectively. However, further increase in the Zr content does not lead to additional resonances as might be expected, and for the lowest level of Sn present (*x* = 0.125) only three signals are still seen, but now at −646, −652 and −658 ppm.

The powder XRD measurements (see above) confirmed the presence of a solid solution and showed no evidence for phase separation into Sn-rich and Zr-rich pyrochlores, in agreement with the DFT predictions in this work. Previous experimental work on La_2_(Sn_*x*_Zr_1−*x*_)_2_O_7_ using ^17^O MAS NMR spectroscopy concluded that the B-site cation distribution was close to random, albeit with some evidence for a weak preference for the formation of Sn–O–Sn and Zr–O–Zr over Sn–O–Zr bonds (*i.e.*, very low levels of Sn and Zr clustering),^32^ suggesting all 13 possible NNN will likely be present to some extent for Sn. This leads to the conclusion that signals from Sn with higher numbers of Zr NNN are overlapped with those arising from 1, 2 or 3 Zr on the surrounding B sites, in agreement with previous work on Y_2_(Sn_*x*_Ti_1−*x*_)_2_O_7_,^3,7^ where multiple resonances were seen in ^89^Y MAS NMR spectra, but the ^119^Sn spectra showed broad and overlapped signals. This behaviour was ascribed to two competing contributions to the isotropic chemical shift: the decrease resulting from the substitution of the less electronegative Ti^4+^ cation, and a concomitant increase arising from the change in cell size on substitution of the smaller Ti^4+^ cation. Although Zr also has a lower electronegativity than Sn (1.33 *cf.* 1.96), Zr^4+^ is much more similar in size to Sn^4+^ (86 ppm *cf.* 83 ppm) than Ti^4+^ (74.5 ppm), resulting in the overlap of some signals in the ^119^Sn MAS NMR spectrum of La_2_(Sn_*x*_Zr_1−*x*_)_2_O_7_, but with better resolution than what was seen for Y_2_(Sn_*x*_Ti_1−*x*_)_2_O_7_. Clearly, however, it would not be straightforward to deconvolute the complex and overlapped lineshapes observed into signals from the 13 (or even just 8) NNN environments, or indeed to predict exactly where each of these signals is likely to appear. Any such analysis also neglects the effects on the shifts from changes in the longer-range structure and/or those in local geometry (*e.g.*, bond angles and bond distances). This makes any analysis of the experimental ^119^Sn MAS NMR spectra, and the extraction of the information they contain on cation disorder, very difficult to carry out without the aid of supporting computational studies.

### 3.3 NMR spectroscopy: simulations

We have performed a computational simulation of the NMR spectra using both the canonical ensemble, as in ref. 7, and the grand-canonical ensemble, as introduced above. For the latter, we have used the equation:10
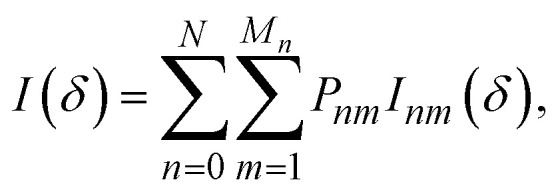
where the configurational NMR intensities *I*_*nm*_(*δ*) are just a superposition of Gaussian functions at each of the DFT-calculated isotropic NMR peaks. The width of the Gaussian (0.85 ppm) was chosen to match the width of the single peak obtained experimentally for *x* = 1. Fig. 5 compares the calculated spectra with the experimental ^119^Sn MAS NMR spectra of La_2_(Sn_*x*_Zr_1−*x*_)_2_O_7_. Although the measurements are done at room temperature, the probabilities in eqn (10) are not calculated at room temperature, because cation redistribution and configurational equilibrium only occurs at elevated temperatures. Instead, we used a configurational temperature of 873 K, which represents an approximate closure temperature below which the cation distribution is frozen when cooling down from the synthesis temperature to room temperature. We have not attempted to estimate a precise closure temperature, which depends on the activation barrier for cation diffusion and is very sensitive to defects in the material (which are not included in our simulations), but our results here are not significantly affected by the choice of configurational temperature within a sensible range, *e.g.* between 600 K and 1000 K. Both ensembles provide a similar picture for high Sn concentrations, *i.e.*, for values of *x* of 0.625 and above the spectra calculated with the canonical and grand canonical ensembles are roughly similar. Larger differences, however, are observed at the lowest Sn concentrations.

**Fig. 6:**
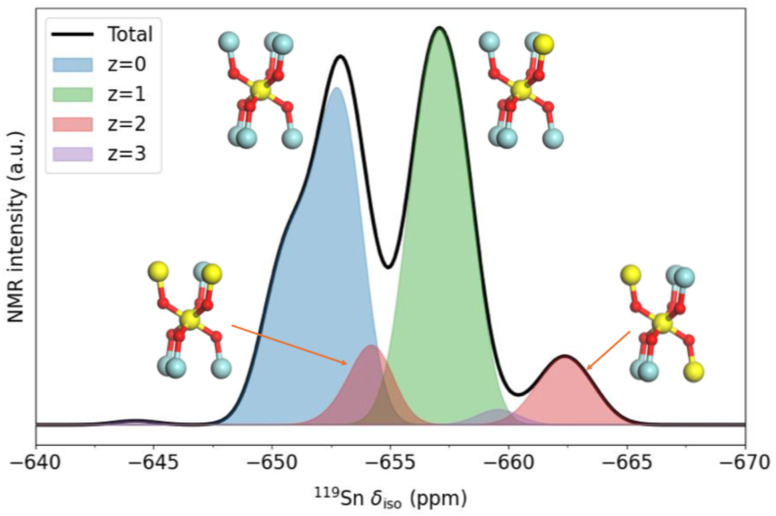
Decomposition of the ^119^Sn NMR spectrum of La_2_(Sn_0.125_Zr_0.875_)_2_O_7_ solid solution (*x* = 0.125), simulated using the grand-canonical ensemble approach, into contributions from given numbers (*z*) of NNN Sn atoms. In the coordination schemes, Sn atoms are yellow, Zr atoms are blue, and O atoms are small and red.

To understand the difference at low Sn concentrations, let us focus on the case with *x* = 0.125 (Fig. 6). In the canonical ensemble simulation, because the cell employed only has two Sn atoms, the spectrum for a configuration with that composition can only show two peaks, corresponding to Sn with *z* = 0 and *z* = 1 Sn NNN, respectively. However, by using the grand-canonical ensemble, we can account for all contributions for any *z* between 0 and 6. The Sn cations with *z* = 0 contribute one of the two large peaks, at values between −647 ppm and −655 ppm (this peak has a small shoulder at *ca.* −651 pm, which results from lower-probability *z* = 0 configurations with higher Sn occupancies in the coordination spheres beyond NNN; that distinction seems exaggerated by our model, as it appears unresolved in experiment). The second large peak, between −655 ppm and −660 ppm, is the *z* = 1 peak, which is present in both the canonical simulation and in experiment. Crucially, in the grand-canonical simulation we also observe that a third peak appears at more negative chemical shifts, between −660 ppm and −665 ppm. That peak is very clear in the experimental spectrum but could not be obtained with the canonical ensemble approach. The peak is the result of clusters of 3 Sn atoms, where a given Sn atom has *z* = 2 NNN Sn atoms, as shown in Fig. 6. In particular, the peak between −660 ppm and −665 ppm appears when an Sn atom has two NNN Sn atoms in a “*trans*” configuration (*i.e.* occupying opposite positions around the central Sn atom in the coordination environment scheme in Fig. 1b). Sn atoms with two NNN Sn atoms in “*cis*” configuration (both NNN Sn atoms in adjacent positions) contribute a peak that appears at less negative chemical shifts, roughly in between the peaks with *z* = 0 and *z* = 1; that overlap makes the *z* = 2 “*cis*” peak difficult to resolve in experiment, in contrast with the *z* = 2 “*trans*” peak, which is clearly visible (Fig. 5a, bottom).

Clusters of 3 Sn atoms are absent in the canonical representation of the composition with *x* = 0.125 for the simulation cell size used in this work, since in this case only 2 Sn atoms are included per cell, and therefore all *z* ≥ 2 contributions are missing. In principle, modelling this compositional fluctuation in the canonical approach is possible, but it would require a larger simulation cell. For example, doubling the cell in only one direction (which would be a minimal but not ideal extension as it would break the cubic symmetry) would give an *N* = 32 supercell, where the composition *x* = 0.125 would be represented by *n* = 4 Sn atoms and therefore *z* = 2, 3 clusters would be possible. However, such large cells quickly become impractical (in terms of computational cost), both because of the combinatorial explosion of possible configurations and the increased computational cost of optimising and predicting the NMR parameters for each configuration in the ensemble at DFT level.

The grand-canonical approach thus provides an elegant route to account for compositional fluctuations in atomic local environment in the simulation of the NMR spectra of solid solutions, without the need for prohibitively large simulation cells, as evidenced in the case of the *x* = 0.125 composition. Still, there are substantial discrepancies between model and experiment at other (mainly intermediate) compositions. For example, at *x* = 0.375 and *x* = 0.5 the simulated spectra seem to have more peaks than those observed experimentally. We believe the discrepancies between model and experiment are mainly due to the factors described below.

First, while the DFT-GIPAW approach is extremely powerful and valuable, it is still approximate and we find that the absolute values of the chemical shifts, as well as some relative values, are not well reproduced. This pyrochlore oxide solid solution is a particularly challenging case as the shift differences are quite small and therefore difficult to resolve. At intermediate concentrations, when the number of possible chemical environments contributing to the spectra becomes higher, prediction errors accumulate more densely, leading to less well-defined peaks and stronger discrepancies with experiment.

Second, while the grand-canonical approach corrects the leading finite-size error of the canonical model in terms of configurational statistics (in the sense that it allows for a more flexible composition of the first Sn–B coordination sphere, regardless of the overall composition), it still suffers from some finite-size effects. The second and third Sn–B coordination spheres correspond to distances (between 6.5 and 7 Å, and between 7.5 and 8 Å, respectively) that are longer than half the length of the simulation cell, thus distorting somewhat the statistical weighting of the smaller but still important longer-range contributions. This effect also accumulates more problematically at intermediate compositions compared to near the solid solution's end-members, further contributing to discrepancies with experiment.

Third, the ion dynamics in the flat potential energy surface of this pyrochlore is not accounted for by our use of (*T* = 0 K) optimised geometries. In our “static” approach, each DFT simulation is performed at a specific local relaxation pattern corresponding to the energy minimum for the given Sn/Zr occupancy configuration. However, we have observed that DFT predicts shallow minima for this solid (to the extent that the precise DFT-optimised local geometries are often sensitive to the details of the optimisation algorithm). Finite-temperature measurements might therefore be influenced by dynamic effects that are not well accounted for in our model.

There are other potential sources of error. For example, chemical shift anisotropies (CSAs) were ignored in our analysis. They are between 60 and 70 ppm, which is about 10 kHz at 9.4 T. Since we spin the samples at 14 kHz, we would expect CSA to have a very small effect. There is also the possibility that the experimental samples have defects in concentrations high enough to affect the NMR spectra, as well as other complexities resulting from kinetic effects in the synthesis. With the computational tools we are developing these effects would be a good area for future investigation, but in the present case they are unlikely to be more important than the more critical simulation limitations described above.

We have also examined the potential contribution of stress-volume effects. In the current formulation, each configuration (*n*, *m*) contributes to the ensemble in proportion to the probability *P*_*nm*_ of eqn (2), with an energy calculated *via* a DFT full relaxation of geometry and cell parameters under periodic boundary conditions. However, if the equilibrium cell parameters vary strongly with composition, one can expect that configurations with *n* far from *xN* would require an additional energy penalty, due to the stress of accommodating the compositional fluctuation, leading to them having lower probability of occurrence with respect to the uncorrected grand-canonical formalism. We have implemented an approximate (volumetric) correction for this effect: if we know both the equilibrium cell volume *V* and bulk modulus *B* as a function of *x*, the energy penalty added to each *E*_*nm*_ as a stress-volume correction (SVC) is:11
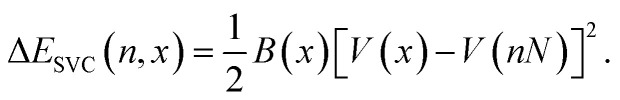


For our La_2_(Sn_*x*_Zr_1−*x*_)_2_O_7_ case study, using bulk moduli and cell volumes obtained from linear interpolation between the DFT values of the endmembers, we found that this SVC correction had no significant effect on the results, which can be explained by the similar radii of Sn and Zr ions and the weak variation of volume with composition in this system. However, the correction given by eqn (11), or more sophisticated versions of it, might be needed in other systems where stress associated with compositional fluctuations plays a more important role.

A final caveat is that, while the grand-canonical formalism allows the adoption of a relatively small simulation cell, it nevertheless still requires a very significant computational effort. Even when one is interested in modelling a specific solid solution composition, it is necessary to calculate energies and NMR parameters for all the configurations at different cell compositions. The number of configurations scales exponentially with the supercell size (as 2^*N*^ for a binary solid solution) and can only be slightly reduced by exploiting the crystal symmetry. We therefore need to explore routes to accelerate the simulations for this computation method to become a realistic analysis tool.

### 3.4 Accelerating the calculations: truncating ensembles

A clear route to decrease the overhead in computational cost associated with the use of grand-canonical ensemble is to limit the calculations to cell compositions near the target one, because configurations for which *n*/*N* is very different from *x* contribute with very low probabilities. If one is interested only in a specific composition *x*, there is no need to consider configurations with the whole range of *n* values, but only those for which *n*/*N* is close to *x*.

**Fig. 7:**
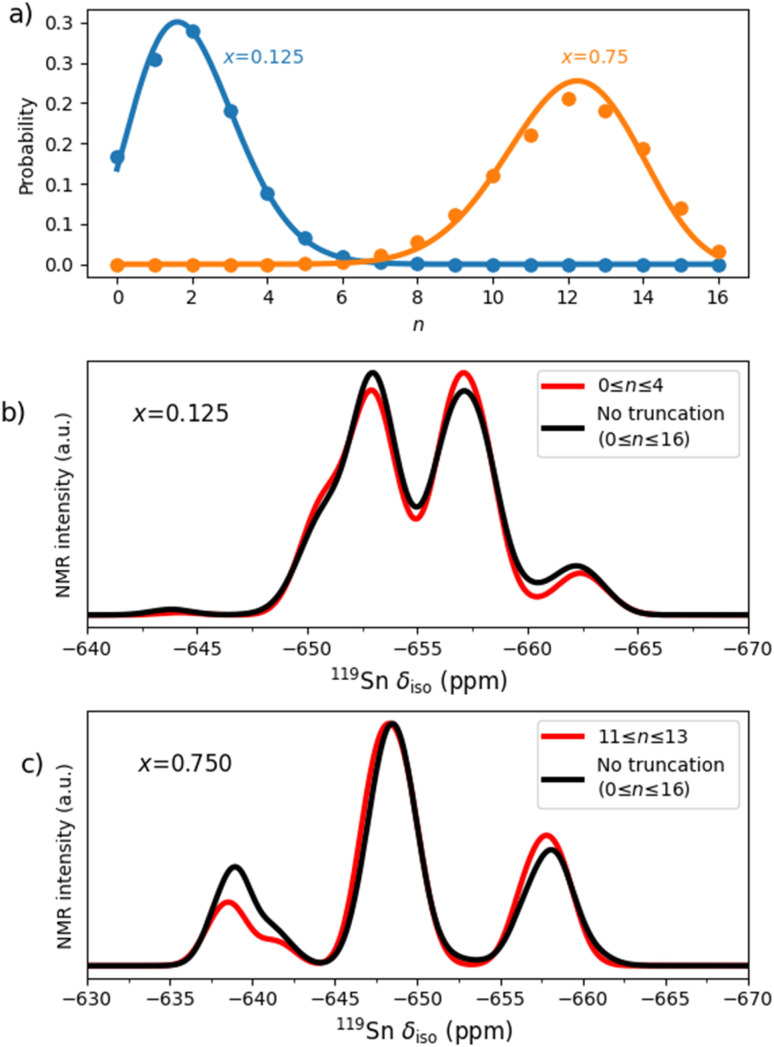
(a) Cumulative grand-canonical probabilities (sum of probabilities over all the configurations at each composition), calculated at 873 K for two La_2_(Sn_*x*_Zr_1−*x*_)_2_O_7_ compositions, *x* = 0.125 and *x* = 0.750. The solid line is given by the binomial distribution and corresponds to the limit of full disorder. (b) Comparison between the ^119^Sn MAS NMR spectrum for *x* = 0.125 obtained using all configurations (0 ≤ *n* ≤ 16) in the grand-canonical ensemble, and that obtained from a truncated ensemble with 0 ≤ *n* ≤ 4 only. (c) The same comparison for *x* = 0.750, featuring the full ensemble and a truncated ensemble with 11 ≤ *n* ≤ 13 only.

We can illustrate this idea with the example of the pyrochlore with composition La_2_Sn_0.25_Zr_1.75_O_7_, which corresponds to *x* = 0.125. In the canonical ensemble, this corresponds to *n* = 2 Sn/Zr substitutions in the cell with *N* = 16 sites. Configurations with other values of *n*, which are omitted in the canonical ensemble, are expected to be relevant as this is a small cell, and we are close to an endmember. Fig. 7a shows the cumulative probabilities 
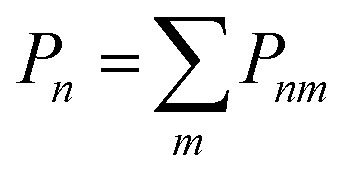
, calculated using the grand-canonical ensemble, at 873 K, as well as those expected in the limit of full disorder (formally corresponding to infinite temperature, but obtained with the binomial distribution) at the same compositions. Because the system is highly disordered, the grand-canonical cumulative probabilities follow the binomial distribution well. As expected, for composition *x* = 0.125 the maximum probability lies close to *n* = 2 substitutions over the 16 B sites, whereas for composition *x* = 0.75 the maximum probability lies close to *n* = 12 substitutions over the 16 sites. The cumulative probability of configurations with a given *n* becomes much lower the farther *n* is from *xN*. We can therefore truncate the number of configurations employed in the grand-canonical analysis, by retaining only configurations with *n* close to the canonical values. In fact, it seems that it is often sufficient to take one or two *n* values at each side of *xN* to achieve a converged result. In Fig. 7b and c, we show the effect of performing the truncated grand-canonical analysis, where instead of using the full range of configurations (0 ≤ *n* ≤ 16), we use only 0 ≤ *n* ≤ 4 (*i.e. n* = *xN* ± 2) for *x* = 0.125 and 11 ≤ *n* ≤ 13 (*n* = *xN* ± 1) for *x* = 0.75. The spectra obtained using these truncated ensembles are very similar to those obtained using the full range of configurations, but they are obtained at a fraction (∼7% and ∼23% in the two cases discussed) of the computational cost of evaluating the full ensemble. The advantage introduced by ensemble truncation seems higher for compositions near the solid solution endmembers.

### 3.5 Accelerating the calculations: machine learning

We have investigated a further route for reducing the time needed to obtain an accurate prediction of the NMR spectra using ML techniques, with the aim of carrying out the minimum number of DFT calculations to be able to simulate complete ^119^Sn NMR spectra. The idea here is to perform only a limited number of DFT simulations, for a selected number of configurations, and use them to train a ML model to predict the rest, avoiding both geometry optimisation and DFT-based NMR parameter computation for the remaining configurations.

We considered five different ML models, for which the results are shown in Table 2, comparing the mean absolute errors (MAE) between the DFT-calculated ^119^Sn chemical shifts and those predicted with ML. The number of configurations included in the training set is 20% of the total number of configurations (we took all the configurations with extreme compositions, *i.e.*, *n* = 0, 1, 2, and 16, 15, 14, plus a random selection from the rest of the compositions to complete 20%). The MAE was evaluated for all the other configurations not in the training set (*i.e.* 80% of the configurations in the ensemble). The best performing model was the random forest, with a MAE just below 3 ppm.

**Table 2:** Mean absolute errors (MAE) of the predicted ^119^Sn isotropic chemical shifts (relative to those obtained using DFT), for the prediction algorithms tested using a training set size of 20% of the total data

Model	MAE (ppm)
Random forest	2.99
Gradient boosting	3.04
SVR	3.37
Bayesian ridge	3.88
Linear regression	3.88

**Fig. 8:**
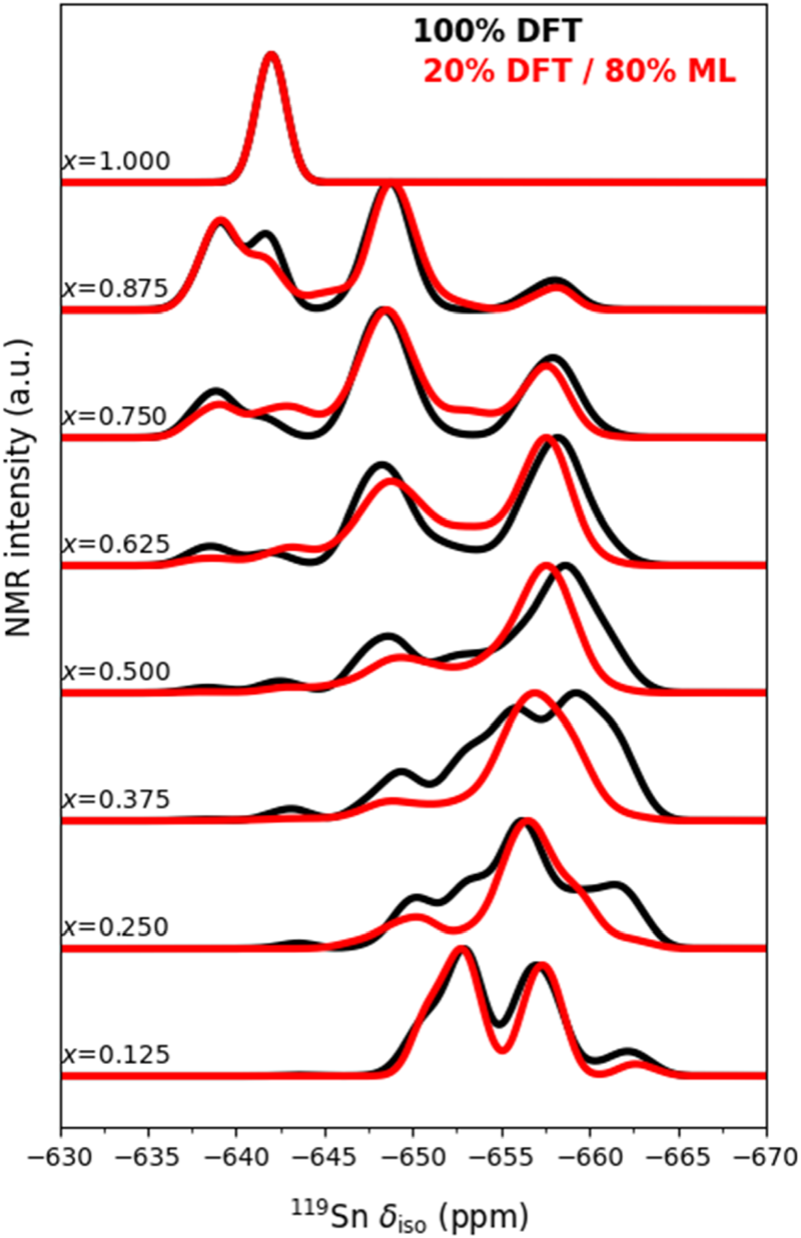
Simulated ^119^Sn NMR spectra of La_2_(Sn_*x*_Zr_1−*x*_)_2_O_7_ obtained using a grand-canonical approach with DFT calculations for all the arrangements in the configurational space (black lines) and predicted with the random forest ML model (red lines), using 20% of the configurations for training.

Since the DFT optimisation and NMR calculations of all the configurations in the full ensemble are so computationally demanding (in our case, *ca.* 40 million CPU hours), if the ML predictions could give results of similar quality to those from the DFT calculations, we would have a method for more efficient prediction of NMR spectra of solid solutions with DFT quality in a much shorter time scale. To explore this, we calculated the ^119^Sn MAS NMR spectra, with the grand-canonical method, using both the DFT-calculated ^119^Sn isotropic shifts, and those predicted with the random forest ML model. As Fig. 8 shows, the spectra calculated with the ML approach shows close similarity with the one calculated in the full-DFT approach. Most of the peaks that appear in the full-DFT simulations are also present in the ML-accelerated simulations, and they are present at the same, or only slightly shifted, chemical shifts.

Still, there is plenty of room for improvement in the ML model. For some compositions the discrepancies with the full-DFT calculations are significant: for example, for *x* = 0.375 the ML-extended model predicts two well-defined peaks, whereas the full-DFT spectrum has a more much complex structure. Interestingly, the ML-aided result at this composition looks more like the experimental spectrum (in Fig. 5) than the full-DFT result does. The reason for this error cancellation is likely to be related to dynamic effects, which, as mentioned earlier, lead to effective averaging over different local relaxation patterns in the experimental spectra: the ML simulation ends up being closer to experiment by avoiding local relaxation effects (it uses the geometry of the unrelaxed lattice to save the cost of DFT geometry optimisations, and that might resemble the dynamic average better than the DFT local minima). This hypothesis deserves further investigation in future studies, perhaps by combining molecular dynamics with ML interatomic potentials and fast ML-aided NMR predictions. In any case, the limitations of the current ML model to reproduce the target NMR chemical shifts from DFT still need to be addressed. For example, we might need to use more expressive descriptors of local chemical environments, such as SOAP (Smooth Overlap of Atomic Positions) feature vectors,^37^ which have been successfully used for NMR predictions elsewhere;^38–41^ and it might be possible to improve our sampling strategy in the creation of the training dataset, including by using active learning approaches.^42^

Despite its limitations, our initial ML results already suggest that conducting the computationally intensive DFT calculations for all configurations may not be necessary if a sufficiently representative training set is available. Instead, a fraction of the original number of DFT calculations (∼20%) appears to be sufficient in this case. Furthermore, our initial results show how future studies could be expanded to include larger simulation supercells to account better for the statistical distribution of chemical shifts without the artifacts introduced by periodic boundary conditions.

## 4 Conclusions

In this work, we have demonstrated that combining DFT calculations with statistical mechanics in a grand-canonical ensemble approach provides an accurate, efficient and flexible framework for modelling local structures in disordered solid solutions. By applying this methodology to ^119^Sn NMR spectra of La_2_(Sn_*x*_Zr_1−*x*_)_2_O_7_, we have shown that conventional single-composition models in a canonical ensemble fail to capture the full complexity of local chemical environments. Specifically, canonical models do not account for compositional fluctuations at the scale of the simulation cell. Given that for quantum-mechanical estimation of NMR chemical shifts we are constrained to relatively small simulation cells, this limitation becomes an important one, leading to clear disagreements with experiment. The grand-canonical ensemble approach naturally resolves this limitation by relaxing the constraint of using simulation cells with the same composition as the overall solid solution, leading to improved agreement between computationally predicted and experimentally observed NMR spectra.

The combination of grand-canonical ensembles and DFT simulations is, however, a computationally expensive approach to the simulation of the NMR spectra of solid solutions. We have demonstrated that ensemble truncation strategies significantly reduce the computational cost of these simulations without significantly sacrificing accuracy, if specific compositions are being studied. Machine learning techniques allow further enhancement in efficiency by decreasing the number of necessary DFT calculations and predicting NMR chemical shifts for uncomputed structures. Furthermore, these techniques open the possibility of performing future studies in larger simulation cells, to afford a more complete description of the configurational statistics of chemical shifts.

The presented methodology not only refines the interpretation of experimental solid-state NMR spectra but also establishes a scalable and transferable framework for studying other complex solid solutions.

## Author contributions

R. G. C., D. M., and S. E. A. conceived the project; S. H. and R. G. C. implemented the method in the SOD code; S. H. also developed additional scripts for data analysis and figure preparation; S. R. G. B. developed the code for extracting local environment features; S. R. G. B., R. I., and T. D. S. carried out the machine learning analysis, with input from S. H. and R. G. C.; D. M. performed the first-principles calculations using CASTEP; A. F., B. L. G., and R. F. M. conducted the experimental measurements under the supervision of S. E. A.; R. G. C., S. H., and S. E. A. drafted the manuscript. All authors contributed to the interpretation of the results and to the final version of the manuscript.

## Conflicts of interest

There are no conflicts to declare.

## Data Availability

The SOD code implementing the grand-canonical ensemble and spectra averaging is available at https://github.com/gcmt-group/sod. The DFT-based NMR shift calculations were carried out using CASTEP, a commercial code that is freely available for academic research use in the UK and in some other regions where institutional licenses exist; researchers wishing to reproduce the CASTEP-based parts of the workflow will require access to a licensed installation. The data and codes needed to reproduce the figures in this article are available at https://github.com/shamgom/NMR_Solid_Solutions. The code to extract the descriptors for the ML analysis is available at https://github.com/salrodgom/data_descriptors_NMR_pyro.
